# Alternative Postoperative Analgesia Technique for Peripheral Vascular Revascularization: A Case Report

**DOI:** 10.7759/cureus.41342

**Published:** 2023-07-04

**Authors:** Paulo T Almeida, Ana R Arantes, Erica G Carvalho, Mário M Vieira, Juliana L Cruz

**Affiliations:** 1 Anesthesiology, Hospital de Braga, Entidade Pública Empresarial (EPE), Braga, PRT; 2 Vascular Surgery, Hospital de Braga, Entidade Pública Empresarial (EPE), Braga, PRT

**Keywords:** femoral popliteal bypass, teamwork, surgical dissection, continuous perineural catheter, multimodal analgesia

## Abstract

Peripheral arterial disease (PAD) patients often require surgical peripheral vascular revascularization (PVR), in which postoperative pain management can be challenging. This case report details a 43-year-old female patient with PAD who underwent urgent femoral popliteal bypass with an inverted ipsilateral great saphenous vein and left femoral endarterectomy. Due to contraindications for neuraxial anesthesia and the necessity for continuous anticoagulation, the procedure was performed under general anesthesia (GA) and an unconventional technique with intraoperative perineural catheter (PC) placement to guarantee adequate postoperative analgesia. The surgeon inserted the PC in the vicinity of the femoral nerve under direct visualization before surgical closure. Postoperative analgesia was successfully managed, demonstrating the effectiveness of this approach as part of a multimodal analgesia strategy. This case report suggests that such a technique, supervised by an anesthesiologist and supported by a multidisciplinary team, can provide effective postoperative pain control in PAD patients without interrupting perioperative anticoagulation. Formal protocols for similar procedures can arise, incorporating this analgesic option.

## Introduction

Peripheral arterial disease (PAD) is due to atherosclerosis causing arterial stenosis or occlusion in major vessels supplying the extremities. Symptoms may include intermittent claudication, critical limb ischemia (CLI), or acute limb ischemia (ALI) due to acute thrombosis. If ALI and CLI are not treated promptly, they can lead to limb loss or even death [1]. Surgical peripheral vascular revascularization (PVR) remains the preferred treatment for physically fit patients whose arterial anatomy is unsuitable for endovascular intervention. Endarterectomy and bypass grafting are two common PVR surgical techniques. Most patients with PAD are under antiplatelet drug or anticoagulant therapy [1].

General anesthesia (GA) and regional anesthesia (RA) have both been successfully used for PVR [1]. Peripheral nerve blocks may provide an alternative to GA and regional neuraxial blocks where contraindicated as per international guidelines [2,3]. In our institution, when feasible, the preference is to perform procedures under spontaneous ventilation with epidural anesthesia, spinal block, or a combination of both. We also use an epidural catheter for postoperative pain relief. We present a case in which the neuraxial approach was contraindicated, leading us to use GA and an alternative method for postoperative pain management. 

## Case presentation

We treated a 43-year-old female patient (American Society of Anesthesiologists (ASA) Physical Status Classification System III) diagnosed with grade III (Leriche-Fontaine) ischemia of the lower limb due to a recent occlusion in the left femoropopliteal transition. The patient was proposed for urgent femoral-popliteal bypass with an inverted ipsilateral great saphenous vein and left femoral endarterectomy. At the time of surgery, the patient was taking both acetylsalicylic acid and clopidogrel, having taken the last dose of clopidogrel three days prior.

Due to contraindications for the neuraxial approach, the surgery was performed under GA with standard ASA monitoring. GA induction was achieved with fentanyl (2 mcg/kg), propofol (2 mg/kg), and rocuronium (0.6 mg/kg). We maintained GA with sevoflurane in an air/oxygen mixture.

**Figure 1:**
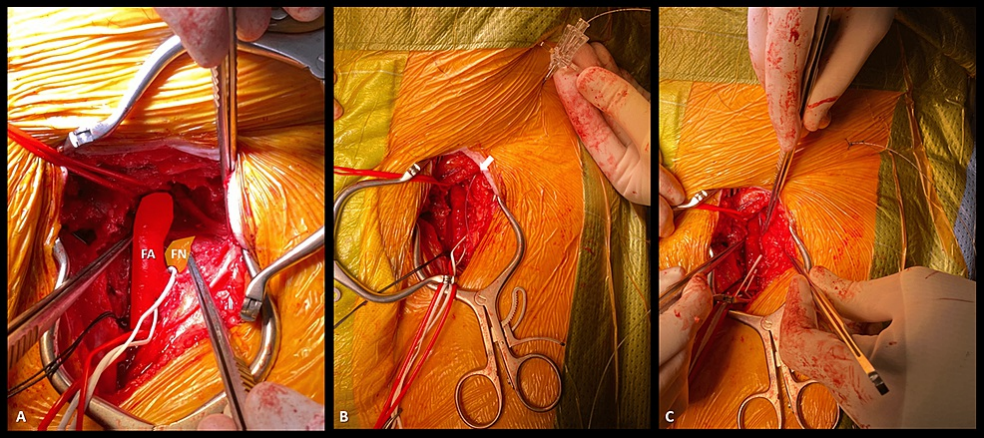
Surgical placement of a perineural catheter in the femoral nerve (A) Identification of the femoral nerve. (B) Placement of the perineural catheter in the femoral nerve, under direct visualization. (C) Fixation of the perineural catheter in the femoral nerve, under direct visualization. FA, femoral artery; FN, femoral nerve; white arrow, perineural catheter

We administered heparin (5000 UI) before applying a femoral clamp. Before surgical closure, the surgeon inserted a perineural catheter (PC) around the femoral nerve under direct vision, leaving it 12 cm from the skin (Figure 1). Ropivacaine (20ml of 0.375%) was administered, and we supplemented it with multimodal analgesia using intravenous (IV) paracetamol (1000 mg) and IV ketorolac (30 mg). Anesthesia has been conducted without any complications.

In the postoperative period, the pain was assessed by the numeric pain rating scale (NPRS). The patient spent two hours pain-free (NPRS 0/10) in the Post-Anesthesia Care Unit and was then transferred to the ward. Postoperatively, the patient reported effective pain control at rest and during mobilization on the NPRS, achieved through a regimen of perineural ropivacaine (10 ml of 0.2% every two hours plus 5 ml if NPRS > 5), IV paracetamol (1000 mg every six hours), IV ketorolac (30 mg every 12 hours for 48 hours), and IV tramadol (100 mg as needed).

Before each ropivacaine administration via the PC, the surgical drain was carefully clamped and released 30 minutes later. In the first 24 hours post-operation, we administered two additional doses of 5 ml of ropivacaine 0.2%. We de-escalated the analgesia on the second postoperative day to 5 ml of ropivacaine 0.2% on demand and only three boluses were needed. The PC was removed on the third postoperative day with no reported adverse effects or complications.

## Discussion

There is currently no sufficient evidence for bypass surgery favoring either RA or GA, as both show no significant differences in long-term survival rates [1]. RA potentially reduces respiratory morbidity and postoperative cognitive dysfunction while providing good-quality postoperative analgesia. However, RA might not be suitable for patients who cannot lie flat due to cardiac, respiratory, or musculoskeletal issues or for lengthy procedures. Neuraxial procedures are also contraindicated for patients on antiaggregant or anticoagulant therapy or who are coagulopathic. For PVR, RA is typically achieved with either spinal anesthesia or a combined spinal-epidural (CSE) technique. CSE techniques are particularly useful for lengthy procedures and when postoperative epidural analgesia is beneficial [1].

However, the widespread use of drugs that affect hemostasis has reduced the number of patients eligible for neuraxial blockade. Peripheral nerve block procedures might offer a suitable alternative for these patients and have been integrated into standard anesthesia practice at many institutions. This strategy partially mitigates the challenge of perioperative anticoagulation and anti-aggregation while ensuring adequate pain control [3]. High-quality analgesia is crucial for patient comfort and to minimize the sympathetic stress response to surgery, which causes tachycardia, hypertension, and vasoconstriction, all detrimental to the patient and graft patency [1,2].

In our case, neuraxial anesthesia was contraindicated, and the procedure was performed under GA. The patient could not discontinue clopidogrel within the necessary timeframe to permit a neuraxial approach. The surgeon inserted the PC into the femoral nerve under direct vision before closure to provide analgesia to the medial leg, in line with the surgical site. The femoral nerve block provides analgesia for the anterior thigh skin, most of the hip joint, femoral periosteum, knee joint, quadriceps muscle, and the skin on the medial side of the leg and foot [4].

Our group has previously published a similar case report with excellent results. In that case, the PC was placed in the adductor canal [5].

Supervised by an anesthesiologist, this technique is a safe option for this surgery because the targeted structures are dissected during the procedure. As part of a multimodal analgesia strategy, this technique allowed for adequate pain control without complications and without interfering with perioperative anticoagulation.

Implementing analgesia by peripheral techniques is only possible when the entire team understands their benefits and risks. In both clinical cases, the nursing team diligently clamped the surgical drain during the administration of local anesthetic and for the subsequent 30 minutes to prevent leakage. A specialized Acute Pain Unit oversaw the patient's follow-up, guiding and managing the analgesic strategy, while the Vascular Surgery Department was responsible for postoperative surgical management. Thus, the success of these strategies depended on a multidisciplinary approach and the total commitment of all team members.

## Conclusions

This case demonstrates the new application of a continuous locoregional technique that leverages the surgical dissection itself and emphasizes the importance of teamwork, with excellent results. Given its simplicity and safety, we believe this analgesic option can be reproduced and serve as the basis for a valid protocol for this procedure. We hope our findings contribute to the ongoing refinement of best practices for pain management in similar surgical contexts.
